# Detection of Genomic Uracil Patterns

**DOI:** 10.3390/ijms22083902

**Published:** 2021-04-09

**Authors:** Angéla Békési, Eszter Holub, Hajnalka Laura Pálinkás, Beáta G. Vértessy

**Affiliations:** 1 Department of Applied Biotechnology & Food Sciences, Budapest University of Technology and Economics, H-1111 Budapest, Hungary; holub.eszter@gmail.com (E.H.); palinkas.hajnalka@ttk.hu (H.L.P.); 2 Genome Metabolism Research Group, Institute of Enzymology, Research Centre for Natural Sciences, Eötvös Lóránd Research Network, H-1117 Budapest, Hungary

**Keywords:** uracil-DNA, dot blot, in situ detection, PCR-based U-DNA detection, genome-wide uracil mapping

## Abstract

The appearance of uracil in the deoxyuridine moiety of DNA is among the most frequently occurring genomic modifications. Three different routes can result in genomic uracil, two of which do not require specific enzymes: spontaneous cytosine deamination due to the inherent chemical reactivity of living cells, and thymine-replacing incorporation upon nucleotide pool imbalances. There is also an enzymatic pathway of cytosine deamination with multiple DNA (cytosine) deaminases involved in this process. In order to describe potential roles of genomic uracil, it is of key importance to utilize efficient uracil-DNA detection methods. In this review, we provide a comprehensive and critical assessment of currently available uracil detection methods with special focus on genome-wide mapping solutions. Recent developments in PCR-based and in situ detection as well as the quantitation of genomic uracil are also discussed.

## 1. Introduction

**Figure 1:**
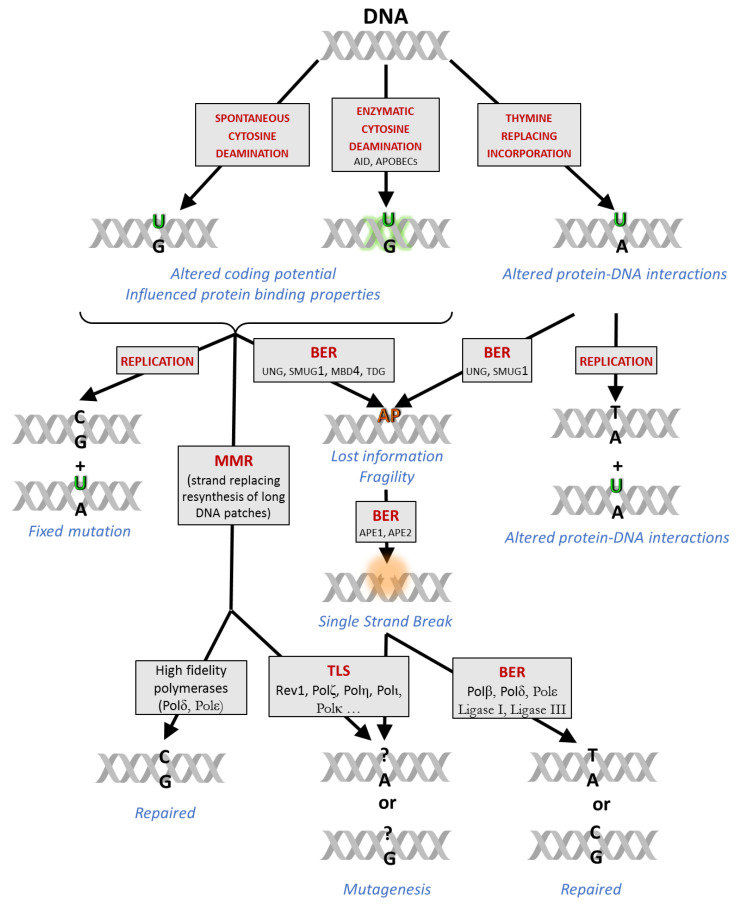
Sources and effects of genomic uracil involving distinct repair mechanisms. Different processes (red letters in grey boxes) are shown with the most important enzymes involved (black letters in grey boxes). Possible consequences of the different outputs are also indicated (blue). Uracil in DNA can arise by spontaneous or enzymatic deamination of cytosines, and by thymine-replacing incorporation. Without repair, U:G mispairs due to cytosine deamination events lead to point mutations right after the next replication, because cytosine and uracil (or thymine) have different base pairing abilities hence different coding potential. Cytosine deamination or the resulted point mutations as well as the “innocent” thymine-replacing uracils (resulted in uracil:adenine (U:A) pairs) can potentially influence interactions between DNA and different DNA binding proteins (such as transcription factors, or polymerases). Uracils can be repaired via base excision repair (BER) initiated by the uracil-DNA-glycosylases: the major enzyme is the uracil-DNA N-glycosylase (UNG); a potential backup is the single-strand selective monofunctional uracil-DNA-glycosylase (SMUG1), while the thymine–DNA–glycosylase (TDG) and the methyl-CpG-binding domain protein 4 (MBD4) can only excise uracils from U:G mispairs. The resulting apyrimidinic/apurinic sites (AP) can be further processed by the AP endonucleases (APE1, APE2). The resulted gap (orange transparent cloud at the single-stranded DNA (ssDNA) break) can be filled correctly by the classic BER polymerases (pol β, δ, and ε) or in error-prone manner by translesion synthesis (TLS) polymerases (Rev1, pol ζ, η, ι, κ, …). The U:G mispairs can be repaired by the mismatch repair (MMR) as well, which involves re-synthesis of long DNA strands usually by high fidelity but sometimes by error-prone polymerases. Both repair processes could result in either mutagenesis or correct repair depending on the recruited polymerases. In a cell, these different steps and ways of repair might occur simultaneously, and their contribution is also influenced by the actual cell cycle phase, the amount of genomic uracils, or the availability of all of these repair factors.

The thymine analogue uracil base has long been considered as an error to be excluded from the DNA; however, key experimental evidence has emerged supporting a more fine-tuned view on the potential impact of genomic uracil (for a detailed review, see [1]). The uracil base (U) can appear in DNA from three sources (Figure 1). First, spontaneous hydrolytic cytosine deamination is one of the most frequent DNA base damage—in a mammalian size genome, several hundreds of such events occur daily, to be compared to the rate of spontaneous depurination (2000–10,000/day/genome) [2,3]. Secondly, DNA cytosine deaminases from the AID/APOBEC family can also catalyze the conversion of cytosine (C) to U in the genome [4,5,6,7,8,9,10,11]. Thirdly, in addition to these potentially mutagenic cytosine deamination events, replicative polymerases can efficiently incorporate 2′-deoxyuridine 5′-monophosphate (deoxyuridylate, dUMP) moieties instead of the naturally occurring 2′-deoxythymidine 5′-monophosphate (deoxythymidylate, dTMP), when 2′-deoxyuridine 5′-triphosphate (dUTP) precursor is abundant, e.g., upon perturbed nucleotide metabolism [12,13,14,15].

Cellular processing and physiological consequences of the genomic uracils are highly context-dependent (Figure 1). Cytosine deaminations result in uracil:guanine (U:G) mismatches that can be fixed as point mutations upon the next replication in one of the daughter cells unless repaired by base excision repair (BER) or mismatch repair (MMR) mechanisms. Thymine-replacing incorporation is less harmful because the DNA coding potential is not changed, however, the missing methyl label from the uracil base as compared to thymine (T) might result in the perturbation of certain DNA binding regulatory functions [16]. Recently, genomic uracil was suggested as a potential epigenetic marker [1,17].

Both the overall amount and the genome-wide distribution of uracils contribute to cellular effects in a strongly context-dependent manner, influenced by numerous factors (Figure 1). For example, mutagenesis due to an increased number of unrepaired deaminated cytosines could be harmful for cell viability [18], but could also potentially lead to malignant transformation. However, limited and strictly regulated C to U deamination events by the activation-induced cytidine deaminase (AID) targeted to the immunoglobulin (Ig) encoding genomic loci [19] are indispensable to trigger essential processes in adaptive immunity such as somatic hypermutation (SHM) and class switch recombination (CSR) [20,21]. Other apolipoprotein B mRNA-editing enzyme catalytic polypeptide-like (APOBEC) enzymes are involved in antiviral defense of the innate immune system [10,22], but overexpression and/or mistargeting of APOBEC deaminases may also lead tumorigenesis, tumor progression, and drug resistance [10,23,24,25,26].

Another particular example for uracil appearance in DNA occurs upon drug treatments by targeting thymidylate biosynthesis, where the massive thymine-replacing uracil incorporation and/or its consequently activated repair by futile cycles eventually lead to cell cycle arrest, cellular senescence, or apoptosis [27,28,29]. The thymineless cell death pathway has been investigated for many years and has been exploited in widely used anticancer therapeutic strategies; however, the molecular mechanism and the exact causative factors have not yet been clearly elucidated.

The main repair pathway for uracil (BER) is initiated by uracil-DNA glycosylases (UDGs) that hydrolyze the glycosylic bond between the base and the deoxyribose providing apyrimidinic/apurinic sites (AP sites) for AP endonucleases (APE1 and APE2) [30,31]. The task to recognize and cleave the uracil base from DNA is shared by four different UDGs in mammalian cells depending on the actual cellular and molecular context. The major enzyme termed uracil-DNA-N-glycosylase (UNG) can efficiently process both U:A and U:G as well as U in ssDNA [32,33]. It has both nuclear (UNG2) and mitochondrial (UNG1) isoforms; the latter provides the sole UDG activity controlling uracil content of the independently replicated mitochondrial DNA [34]. UNG can also recognize further uracil-derived non-canonical bases, e.g., 5-fluoro-uracil, hydroxyuracil, isodialuric acid, and alloxan [33]. The single-strand selective monofunctional uracil-DNA-glycosylase (SMUG1) prefers ssDNA in vitro [35], however, it can also constitute an efficient backup for UNG within the cellular context [33]. SMUG1 recognizes and processes formyluracil, hydroxyuracil, and hydroxymethyl-uracil (hmU) bases, as well [36,37].

In contrast to UNG and SMUG1, the uracil excision activities of the other two UDGs are confined to U:G mispairs. Thymine–DNA glycosylase (TDG) can efficiently remove not only U but also T and various 5-substituted uracil derivatives once they are mispaired with G [38,39,40]. TDG is also implicated in epigenetic and developmental processes via its contribution to active DNA demethylation [41,42,43]. The methyl-CpG-binding domain protein 4 (MBD4) is also specialized for thymine analogues in mispaired context, and its activity has been implied in epigenetic remodeling processes at CpG islands [38,44]. TDG and MBD4 have a key role in the interplay between BER and MMR to repair U:G mismatches [45].

AP sites represent a potentially mutagenic information-lost-state (e.g., when processed by translesion synthesis (TLS) polymerases). If present at a high level, AP sites might influence essential cellular processes such as replication and transcription. The BER pathway is normally completed by resynthesis of the single missing nucleotide, or a longer piece of DNA replacing the original DNA strand downstream, followed by ligation of the single-strand break and also removing the 5′-deoxyribose phosphate (5′dRP) or the longer flanking DNA track [46,47]. The repair synthesis is mostly mediated by polymerases, such as polymerase β in short patch BER, and either one of polymerases β, δ or ε in long-patch BER. However, during SHM, error-prone polymerases (e.g., REV1 and pol η) are also implicated in the concerted action of BER and MMR [45,48,49]. In general, the choice of the polymerase depends on the actual availability and post-translational modifications of different polymerases that is also regulated at multiple level (e.g., by cell cycle, or DNA-damage response (DDR)) [50]. The numerous different steps and routes of repair occur simultaneously within the cell and need to be correctly considered in experimental design and interpretation of results.

**Figure 2:**
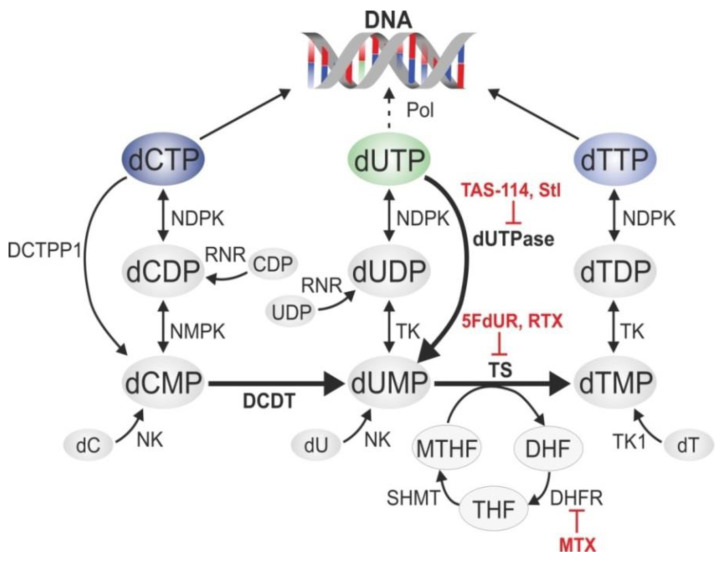
Pathways and potential target enzymes in thymidylate biosynthesis in eukaryotes. Thymidylate synthase (TS) produces deoxythymidylate (dTMP) from deoxyuridylate (dUMP). Inefficient catalysis of this process leads to a decrease in the cellular dTMP level that can result in deoxyribonucleotide triphosphate (dNTP) pool imbalance and perturbed DNA replication. Thymidylate synthase inhibitors raltitrexed (RTX) and 5-fluoro-2′-deoxyuridine (5FdUR) are indicated. For efficient TS activity, the 5,10-methylene tetrahydrofolate (MTHF) co-substrate is indispensable that is recycled from dihydrofolate (DHF) in the folate cycle involving dihydrofolate reductase (DHFR) and serine hydroxymethyltransferase (SHMT) via a tetrahydrofolate (THF) intermediary. Antifolate derivatives (e.g., methotrexate (MTX)) inhibit the folate cycle. Furthermore, production of the precursor dUMP is ensured by two processes: catalyzed by deoxyuridine triphosphatase (dUTPase) and dCMP deaminase (DCTD) together with deoxycytidine triphosphatase (DCTPP1). dUTPase inhibitors TAS-114 and Stl are also shown. Further abbreviations are as follows: nucleoside-diphosphate kinase (NDPK), ribonucleotide reductase (RNR), nucleoside-monophosphate kinase (NMPK), nucleoside kinase (NK), thymidine kinase (TK1), dTMP kinase (TK), DNA polymerase (Pol).

The biosynthetic pathways of pyrimidine nucleotides have evolved to keep an extremely low cellular dUTP/dTTP ratio to minimize the frequency of thymine-replacing uracil incorporation (Figure 2) [1]. Within this network, some enzymes show strict substrate specificity, such as dCMP deaminase (DCTD) [51,52], deoxyuridine triphosphatase (dUTPase) [53], and thymidylate synthase (TS) [54], which are dedicated to efficiently catalyze specific reactions producing dUMP and dTMP, respectively (Figure 2). Other enzymes in these pathways have a wider substrate pool: nucleoside-diphosphate kinase (NDPK), nucleoside-monophosphate kinase (NMPK), and dTMP kinase (TK) act on nucleotides containing different bases. Nucleotide kinases (NK and TK1) are involved in salvage pathways utilizing 2′-deoxynucleosides from external sources to produce the corresponding 2′-deoxynucleotide-monophosphates. The folate cycle (involving dihydrofolate reductase (DHFR) and serine hydroxymethyltransferase (SHMT)) is essential to recycle methylene-tetrahydrofolate (MTHF), the co-substrate of TS that serves as a methyl donor for dTMP synthesis.

In thymidylate biosynthesis, TS plays a key role and its inhibition causes massive decrease in the cellular dTMP level, leading to an increased dUTP/dTTP ratio and massive uracil incorporation into genomic DNA [53]. Notably, TS also has an RNA binding function, regulating the translation of its own mRNA in a negative feedback loop, and also the mRNA of p53 [55,56,57,58]. Further effects include imbalances in the overall dNTP pool that eventually lead to programmed cell death. Direct targeting of dUTPase by small molecular drugs (e.g., TAS-114 [59,60,61]) or the protein inhibitor Stl also provide a promising possibility for anti-cancer therapies [14,62,63,64,65,66,67,68]. RNAi-directed suppression of dUTPase has been shown to increase cellular sensitivity towards TS-inhibiting drugs [69,70].

Inhibitors of TS and DHFR are among the most widely used anticancer drugs (Figure 2), and a wealth of experimental and clinical evidences is available about the possible mechanisms and their respective efficiency, as well as various sensitizing and resistance factors [71]. Two major mechanisms are involved: fluoro-pyrimidines and their active metabolites efficiently block and modify the nucleotide binding site of TS, while antifolates, being structurally similar to folates, compete with binding of the MTHF co-substrate.

In summary, uracil in DNA can be considered as undesired DNA damage, which also can be exploited in anti-cancer therapeutic strategies. Moreover, genomic uracils can serve as signals possibly involved in crucial biological processes such as immune diversity, antiviral defense, and epigenetic regulation of transcription. To better understand these possible roles, it is essential to apply state-of-the-art methodology for characterizing genomic uracil patterns. In this review, we provide a comprehensive and critical assessment of the repertoire of available methods measuring uracil-containing DNA (U-DNA) in different contexts with a special focus on next-generation sequencing (NGS)-based genome-wide mapping solutions.

## 2. Uracil-DNA Detection Methods

Investigation of DNA damage, repair, and epigenetic base modifications became a rapidly developing scientific field, especially in the last decade, fed by numerous new technical solutions such as a new generation of DNA sequencing approaches [72,73]. Li and Sancar provided a comprehensive overview on crucial methods and developments in the field of genome-wide DNA-damage mapping approaches; however, uracil was not fully covered in their work [72]. Another review from Sturla’s lab focuses on NGS-based DNA damage sequencing methods, providing a thorough categorization based on the different technical solutions for library preparation [73].

Here, we provide a summary of diverse uracil-DNA detection methods with their advantages and limitations, and discuss their results and conclusions. We detail the global quantitative U-detection methods as well as various emerging solutions for in situ uracil-DNA detection. Regarding the genome-wide mapping methods, relevance, and benefits of single base resolution, as well as the potential pitfalls in data analysis, are also considered.

### 2.1. Global Quantification of Uracil in DNA

The most straightforward way to quantify the overall uracil content of a DNA sample is a liquid chromatography coupled to tandem mass spectrometry (LC-MS/MS) method [74]. It is based on enzymatic digestion of the DNA to 2′-deoxy-ribonucleosides using DNase I and nuclease P1, followed by a preparative HPLC purification coupled MS/MS identification of deoxyuridine (dUrd) and employs an isotope labelled internal standard. With this approach, and systematically addressing possible technical pitfalls, the uracil content of the murine and the human genome was determined to be ~0.15 and ~0.08 uracil/10^6^ bases, respectively, considerably lower than suggested previously by other MS-based methods [75,76,77,78]. UNG deficiency led to some increment up to ~1.2 and ~0.35 uracil/10^6^ bases, respectively.

Another approach utilizes alkoxyamine-based aldehyde reactive probes (ARPs) to chemically label the aldehyde group in the deoxyribose moiety at AP sites [79]. Biotinylated ARP reagents were used for the detection of oxidative base damages and AP sites on (ELISA-like) dot blot application [80]. The Ung-ARP assay was developed in Bennett’s group, where specific enzymatic removal of the uracil and detection of the resultant AP sites by biotinylated ARP reagent were combined [81]. Further developments led to two alkoxyamine reagents, AA3 and AA6, associated with increased reactivity and functional groups, appropriate to conjugate with a wide variety of biochemical labels by click chemistry [82,83]. These reagents were used in different applications [84,85,86].

As an independent approach, a new U-DNA sensor protein was developed for multiple purposes, including quick one-step semi-quantitative dot blot application where uracil is directly recognized, without any further enzymatic or chemical reactions [28]. For this, the inactive D145N/H268N double mutant of human UNG was used as a starting construct from which the N′-terminal 84 residues were deleted to eliminate undesired protein–protein interaction surfaces (ΔUNG sensor) [87,88]. It was demonstrated that such UNG-based sensor equipped with 3xFLAG tag is an appropriate tool in dot blot application to quantify uracil as compared to a standard with known uracil content [28]. Uracil levels were determined in both bacterial (wt, *ung*^−/−^, and also *ung*^−/−^ *dut*^−/−^ double mutant *E. coli*), and higher eukaryotic (*Drosophila* S2 cells, as well as human colon cancer cell line HCT116) genomes upon treatments with thymidylate biosynthesis inhibitory drugs. The fast and straightforward dot blot applications do not require mass spectrometry infrastructure; however, the mass spectrometric methods provide higher accuracy, especially at low uracil levels.

### 2.2. In Situ U-DNA Detection Methods

Two early approaches, radio labelling [89] and a modified comet assay [90], were applied for in situ U-DNA detection, allowing quantitation with only low resolution. Recently, the ΔUNG sensor described above was further developed to allow in situ detection as well. First, fluorescently tagged ΔUNG sensors were shown to be appropriate for the in situ detection of uracil-containing exogenous plasmid DNA within the context of eukaryotic cells [28]. Later, this sensor was equipped with SNAP-tags and used in super-resolution fluorescent microscopy (STED and dSTORM) to detect endogenous genomic uracils in human cells [91]. The FLAG-tagged ΔUNG sensor was also used for genome-wide mapping (U-DNA-Seq), and the combination of the two approaches revealed that the RTX and the 5FdUR treatment induced uracil-enriched regions/loci colocalized with the active histone mark, H3K36me3, and the facultative heterochromatin mark, H3K27me3, respectively [91].

The mycobacterial UdgX that forms a covalently trapped complex with uracil-DNA was also employed for U-DNA detection [92]. The UdgX enzyme belongs to a bacterial UDG family harboring an Fe–S cluster, with a sequence motif KIRRH (called R-loop) essential for its function. Three-dimensional (3D) structures of UdgX complexes together with LC-MS/MS analysis revealed a covalent link between His109 of the KIRRRH track and the deoxyribose at the AP site [93,94,95]. It is straightforward to use this unique enzyme as a uracil-DNA sensor, similarly to the ΔUNG described above. An mCherry tagged UdgX was constructed and characterized in detail as a highly sensitive U-DNA sensor for detecting U-DNA by confocal microscopy in wild type (wt) and *ung*^−/−^ *dut*^−/−^ *E. coli* [96]. A FLAG-tagged UdgX-based sensor was also used in human cells to detect uracils in ssDNA arising upon the induction of APOBEC3A in cisplatin-treated HEK293T cells, revealing uracil colocalization with replication protein A in stalled replication forks [85]. An advantage of this sensor might be that it can be applied by transfection into living cells, and then only the immunodetection steps have to be carried out in fixed samples. UdgX demonstrated a strong (3–5 orders of magnitude) preference towards uracils in ssDNA context over U:A in dsDNA that, on one hand, ensures a selectivity, and on the other hand, somewhat limits its applicability for addressing certain biological questions. It is important to note that the SNAP-tagged ΔUNG construct recognizes uracils within fixed cells in all those contexts that are normally recognized by the wt UNG (ssU, U:A and U:G pairs).

These in situ detection solutions provide potent tools within highly different biological samples for relatively quick and efficient detection of genomic uracils either upon their increasing levels (e.g., drug treatments [91]) or upon spatial clustering into genomic loci (e.g., targeted enzymatic cytosine deamination [85]). Earlier, in situ detection method for AP sites were available [77], but their application for detection of genomic uracil is not straightforward at all. With the new direct approaches described above, it is also possible to identify (or even screen for) new biological situations and/or conditions, where particular patterns emerge indicating special potentially novel biological roles of genomic uracil. In combination with genome-wide localization data that could provide good candidate protein markers for in situ colocalization studies, these sensor constructs and the coupled detection strategies might especially be powerful. Such in cell studies are more cost-efficient and flexible for the wide screening of treatment-induced changes in various biological samples as compared to the genome-wide NGS-based sequencing methods. However, without the knowledge of genome-wide distribution, just the in situ detection alone could not provide essential new insight to the diverse roles of genomic uracil. Hence, combination of the two approaches is indispensable.

### 2.3. PCR-Based Methods for Uracil Localization within DNA

To localize uracils within a target DNA sequence, several PCR-based methods are available that can provide either an indication for the presence of U:G pairs, or exact localization (even with single-base resolution), or accurate quantitation (within the target sequence) of the uracils. The first published technique to detect C:G to T(U):A transitions due to cytosine deamination in DNA was the differential DNA denaturation PCR (3D-PCR) [97]. This technique relies on the lower denaturation temperature (Td) of DNA templates with higher AT content. It applies gradiently lowered Td in the PCR reactions, amplifying a specific target sequence defined by the two PCR primers. The specific PCR product could be detected already at lower Td in those cases where some C:G to T:A transitions happened in the template DNA within the amplified region [97]. Later, this 3D-PCR technique was applied in combination with a UNG inhibitor, UGI, to detect uracil-DNA intermediates of APOBEC3A-catalyzed cytosine deamination in a reporter plasmid DNA [22].

Almost at the same time as the publication of 3D-PCR, the Gearhart group applied a combined in vitro reaction of UNG and APE1 to detect uracil in an exogenous plasmid from which AID was expressed in bacteria [98]. They could show AID induction-related increases in the number of nicks by a UNG/APE reaction on alkaline agarose gel, and could locate the uracil moieties on the non-transcribed strand using a denaturing Southern blot. Furthermore, they also applied polymerase β without the addition of dNTPs, just using its 5′dRP hydrolyzing function to introduce nicks to the site of the uracil. Then, they applied primer extension on the nicked template using a specific and biotinylated primer that results in dsDNA end that is appropriate for blunt end adapter ligation. By clonal sequencing of the products of this ligation mediated PCR (LM-PCR), they could localize the original positions of uracils within the non-transcribed strand of the AID-expressing plasmid with single base resolution [98]. Later, they further developed this technique and successfully detected uracils in the immunoglobulin genes of *ung*^−^deficient AID expressing B cells as compared to *ung*^−^deficient *Aicda*^−/−^ cells (*ung*^−^deficient chicken DT40 either overexpressing the chicken AID or *Aicda*^−/−^ clones, and B220+GL7+ spleen cells from *ung*^−/−^ and *ung*^−/−^, *Aicda*^−/−^ mice that were either immunized right before the cells were isolated, or the isolated cells were stimulated ex vivo with LPS and Il-4) [99].

Another PCR-based approach simply quantifies the difference between the amounts of intact templates in the samples pre-treated with UNG alone or UNG + APE as compared to the non-treated one. Such quantification could be performed by qPCR, and also by the more convenient digital PCR techniques (such as digital droplet PCR (ddPCR)). In the ex-ddPCR method, samples are treated with UNG, and the fraction of amplicons containing at least one uracil on each strand is determined from positive PCR counts in the treated and un-treated samples [100]. It was shown that the viral gag gene accumulates uracils only in monocyte-derived macrophages (MDM), but not in T cells.

While these approaches above rely on specific enzymatic reactions by UNG and APE, a fully independent PCR-based method utilizes the altered sensitivity of archaeal DNA polymerase Pfu and its V93Q mutant version for the uracil-containing templates [27]. The structural basis and the functional consequences of binding of the archaeal family B polymerases to uracil bases in the template DNA strand has already been well described [101]. While a single uracil base can eventually stall DNA synthesis by wt Pfu polymerase, V93Q mutant Pfu preserves its activity even on fully uracil substituted templates [102]. Applying wt and V93Q mutant Pfu in parallel PCR reactions using the same template dilution series, from the difference between the corresponding Cq values, the uracil can be quantified within the given template region defined by the two PCR primers [27].

### 2.4. NGS Based U-DNA Detection for Genome-Wide Mapping

All methods described above are PCR-based; hence, they are limited to determination of a local uracil content that can be valid for the whole DNA sample as much as the genome-wide distribution of the uracil is uniform. Depending on the origin of the uracil-DNA, its genome-wide distribution can be more or less patterned: enzymatic cytosine deamination might result in a strongly targeted localization (e.g., focusing on variable and switch regions of the Ig genes, or kataegic-like clusters [25]), while spontaneous cytosine deamination and thymine-replacing misincorporation due to the insensitivity of the DNA polymerases are more stochastic and random processes. In these latter cases, if any pattern exists, it should be originated from several additional mechanisms, such as altered accessibility of the differential packaged genomic DNA, the unequal distribution of repair processes, and different polymerases with altered sensitivity and specificity. Indeed, in the last decade, numerous new results support the hypothesis that distinct repair proteins [103] and/or different polymerases [104] are loaded to certain genomic loci rather differently. Since 2014, many NGS-based approaches have been published addressing epigenetic marks (ChIP-seq), or DNA methylation [105], or DNA repair loci (e.g., XR-seq, HS-XR-seq, and Damage-seq [50,106,107]), or other base modifications (e.g., OG-seq [108], click-code-seq [109]), or AP sites (e.g., snAP-seq [103]). Similarly, genome-wide uracil mapping solutions have been developed and are becoming crucial to better understand the significance and consequences of uracil appearance in DNA within the different biological contexts. For these “seq” methods, the PCR-based uracil localization or quantifying techniques described above provide essential validation opportunities.

**Figure 3:**
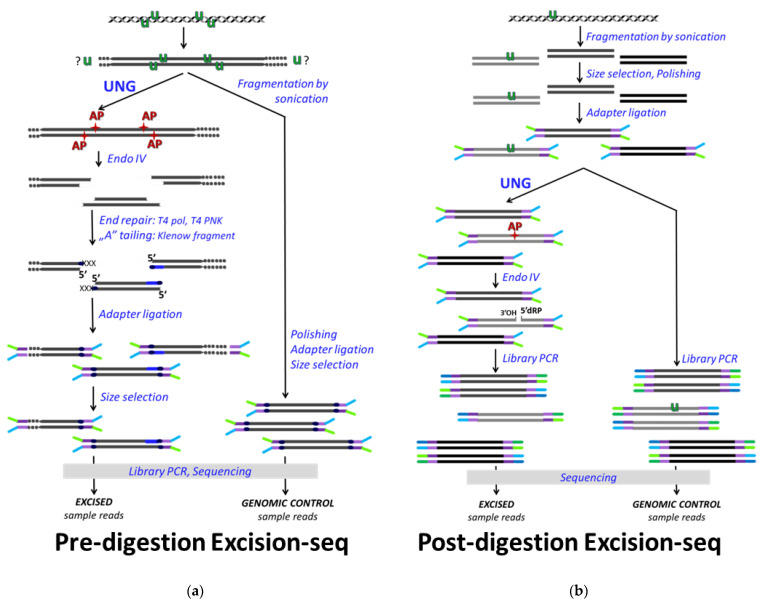
Excision-seq for genome-wide mapping of U-DNA. (**a**) Pre-digestion Excision-seq. For efficient detection, high density of genomic uracils (green U) is required, because NGS-compatible fragments originate from the UNG/ENDO IV treatment. Longer fragments (indicated with dots at the end) are lost during the size selection. End repair involves T4 polymerase (T4 pol), T4 polynucleotide kinase (T4 PNK), and Klenow fragments. Both ends of the sequencing reads report on uracil sites, but some uracils might escape detection. Non-specific hits are filtered by the comparison to genomic control sequencing. (**b**) Post-digestion Excision-seq. Genomic fragments are treated with UNG/ENDO IV, and degradation of uracil-containing fragments results in locally decreased read coverage as compared to the sequencing of the non-treated genomic fragments.

The first published method applied for genome-wide uracil mapping was the Excision-seq applied in *E. coli* and yeast [110]. Excision-seq also operates with the coupled enzymatic reactions of bacterial UNG and the AP endonuclease, ENDO IV, and combines this with massively parallel DNA sequencing (NGS). Two versions had been developed: the pre-digestion (Figure 3a) and the post-digestion Excision-seq (Figure 3b). The pre-digestion version requires high uracil content within the studied DNA sample that allows efficient DNA fragmentation already by the in vitro UNG/ENDO IV enzymatic treatment. Then, applying a size selection without additional fragmentation procedure, the sequencing library is prepared. The ligation position of the sequencing adapter at the 5′ ends will report on the original sites of uracils with practically a single-base resolution (Figure 3a), similarly to the ligation mediated PCR method [99] described above. As a complementary approach, post-digestion Excision-seq applies UNG/ENDO IV treatment on the prepared DNA fragment library, and the increased read coverage in the sequencing results of the excised samples compared to the non-treated controls indicates genomic regions from which uracils were excluded. However, the sensitivity of such inverse approach highly depends on the sequencing depth and requires a rather uniform genome coverage, which might limit the size and the complexity of the genomes addressed by this technique. The two versions of Excision-seq were reported as adequate methods for the efficient detection of elevated uracil levels upon dUTPase and UDG deficiency in smaller genome sizes, as in *E. coli* and yeast strains [110]. They concluded that uracil is excluded from the very early and very late replication timing genomic segments and assumed that such regulation might involve the alterations of the cellular dNTP pool during the DNA synthesis [110]. Nevertheless, a larger genome size (e.g., mammalian genomes) and the low frequency and/or the nature of the distribution of uracils might result in some biases or underestimation using Excision-seq method, especially its pre-digestion version. In this aspect, enrichment or pull-down-based methods might be more efficient. Moreover, it has not yet been demonstrated how beneficial the single base resolution capability of pre-digestion Excision-seq is. Indeed, the same group used this method addressing the 10 kb-size HIV genomes from different in vitro infected immune cells showing uniformly distributed uracilation of the proviral genome [100]. Although this method was also extended to the mapping of other DNA base modifications [111] and cited by reviews or other research papers, Excision-seq has not yet become widely used to characterize other biological systems. In one case, pre-digestion Excision-seq was applied as a complimentary technique to support the results from dU-seq (which will be discussed later [112]).

Meanwhile, also attempting single base resolution detection of uracil (and other DNA lesions) within the DNA, two other approaches were developed on model DNA and proposed to be used in genomic context too by Burrows’ lab [113,114]. Their first method also relies on the UNG/APE enzymatic treatment (or more generally, the other base modification-specific glycosylases and the appropriate AP endonuclease or AP lyase) which is followed by enzymatic labelling of the gapped strand by unnatural nucleotides (dNaM or dMMO2). The bases of these nucleotides are selectively paired with d5SICS unnatural base in PCR reactions forming unnatural base pairs (UBPs). Such UBPs can then be detected either by Sanger sequencing (UBPs stop the seq reactions), or by nanopore sequencing technology, where the position of UBPs can be determined with single nucleotide resolution in the context of single DNA duplexes [113,115]. This method was developed and tested on synthetic DNA models, simulating biologically relevant lesions with their heterogeneous sequence context and also the effect of a large excess of undamaged DNA [113]. It was also suggested that, in combination with an enrichment of DNA lesion-containing DNA-strands, the method can be adapted for complex biological systems. Their second method relies on ligatable gaps that arose upon in vitro treatment by the glycosylase specific for a given modified base; then, sequencing of ligated products by any commonly used NGS techniques and identification of single nucleotide deletions will report on the position of the original DNA lesions [114]. Although this approach seems to be cheaper and more available for the wide scientific community, it has not yet been demonstrated that it could work on large genomes, especially with low uracil content. Basically, the limitations of this method should be similar to those in the pre-digestion Excision-seq. Indeed, the authors suggest that its best application might be in single cell sequencing, where a certain base modification is present at 100% (note: 50% in case of diploid genomes). Furthermore, the relatively high chance of a single nucleotide deletion as a consequence of sequencing error or naturally occurring variation within the sample can also impair the sensitivity.

**Figure 4:**
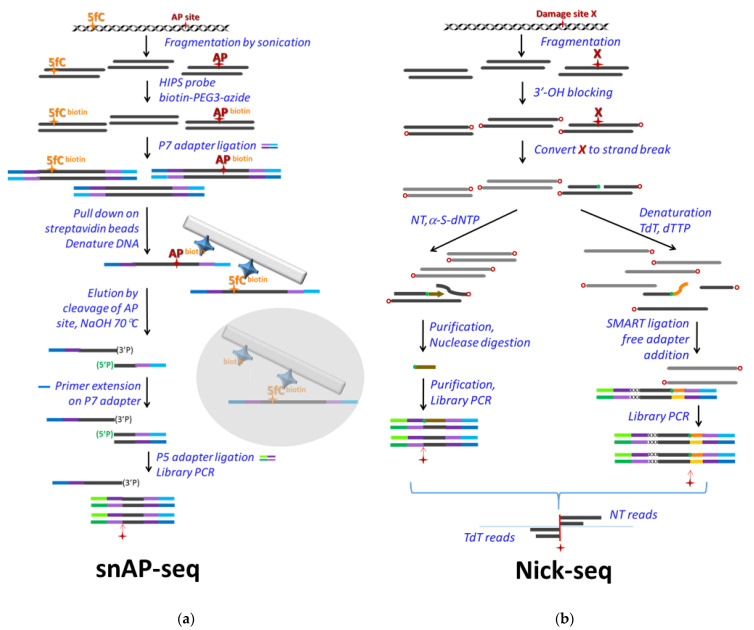
(**a**) snAP-seq. Genomic DNA fragments are chemically labelled by an aldehyde-reactive HIPS probe [116], either at the AP sites (red star) or at formylcytidine (yellow “5fC”) or formyluridine. The HIPS probe is labelled by biotin via a PEG3 linker (biotin–PEG3–azide) which allows efficient pull-down of the P7 adapter ligated and labelled fragments on streptavidin beads (grey rectangle represents the solid phase, blue star: streptavidin tetramers). After denaturation of the dsDNA bound on the beads, AP site fragments are selectively eluted by alkaline cleavage, when only one of the DNA strands downstream from the AP site will be appropriate for ligation. Upon primer extension from the P7 adapter, the resulted dsDNA will also be ligated to the P5 sequencing adapter. The PCR amplified library is sequenced, where the first base of the P5 reads will report on the AP sites (red star). (**b**) Nick-seq. First, 3′-OH groups are blocked by dideoxy nucleotide addition (red “o”) to the fragmented genomic DNA. Then, damage sites (red “X”) are converted to ssDNA breaks with free 3′-OH (green dot). Further processing is performed parallelly in two portions of the sample. NT: nick translation by polymerase I using α-thio-deoxynucleoside triphosphates (α-S-NTP) precursors that will result in phosphorothioate oligonucleotides resistant for nuclease treatment. These purified fragments are used for sequencing adapter ligation, library PCR and sequencing. TdT: terminal transferase is used to tail the free 3′-OH with poly(dT) using the commercially available SMARTER Chip-seq kit (Clontech). Addition of the sequencing adapters then depends on the poly(dT) tail, and applies a template-switching polymerase reaction instead of ligation, ensuring higher specificity of the library preparation. The sequencing results from NT and TdT processing are compared, and reads are positioned next to the damage site downstream and upstream, respectively.

Two other sequencing methods with single base resolutions, which were developed for the detection of AP sites but can easily be adapted for uracil detection by applying preceding UNG treatment (as is also true for other seq methods designed for AP sites), are also worth being presented here. In Balasubramanian’s lab, snAP-seq was developed and used in different size of genomes and for answering different biological questions [103] (Figure 4a). snAP-seq applies a selective chemical labelling of AP sites [116], and enrichment via a biotin–streptavidin system. They demonstrated the selectivity of their method for AP site aldehyde over the formylcytosine aldehyde, via combination of the chemical labelling of the aldehyde groups and the elution from the streptavidin resin using an alkaline condition that hydrolyzes the sugar–phosphate backbone at the AP site. First, chemical labelling is performed on the fragmented DNA, then the P7 sequencing adapter is ligated, followed by the pull-down on streptavidin beads and selective elution by alkaline cleavage. Using the P7 adapter, a primer extension is performed on the eluted ssDNA fraction, then only the AP cleavage-related 5′-phosphates are available for ligation with the other sequencing adapter, P5. Hence, the enrichment of relevant DNA fragments is quite efficient, and a majority (95%) of the sequenced fragment will start exactly one base downstream the original AP sites, as it was measured in a model DNA. This method was applied for single base resolution detection of hmU in *Leishmania major* [103], where hmU is a precursor of the epigenetic marker base J and is supposed to be introduced enzymatically [117,118,119]. The method was also used in human cell lines to detect AP sites upon the silencing of APE1; however, its single base resolution potential could not really be exploited in this latter case, due to more randomized genomic distribution of the AP sites [103].

The other similarly creative method is Nick-seq, developed by Dedon’s group for single-nucleotide resolution genomic maps of different DNA modifications and damage [120] (Figure 4b). The method relies on conversion of the modified bases into single strand breaks on which two different types of polymerase reactions are performed separately. One portion of the sample is subjected to nick translation using α-thio-dNTPs to produce hydrolysis-resistant oligonucleotides downstream of the single strand break. Hence, the rest of the DNA can be selectively removed by exonuclease III and RecJ, and the resistant phosphorothioate-containing oligos can be sequenced. The second portion of the sample is used for poly(dT) tailing of the 3′ end at the strand break by terminal transferase (TdT). Then, this tail is used for library preparation. By this approach, sequencing of the two separately processed samples can confirm the position of a base modification from two directions.

**Figure 5:**
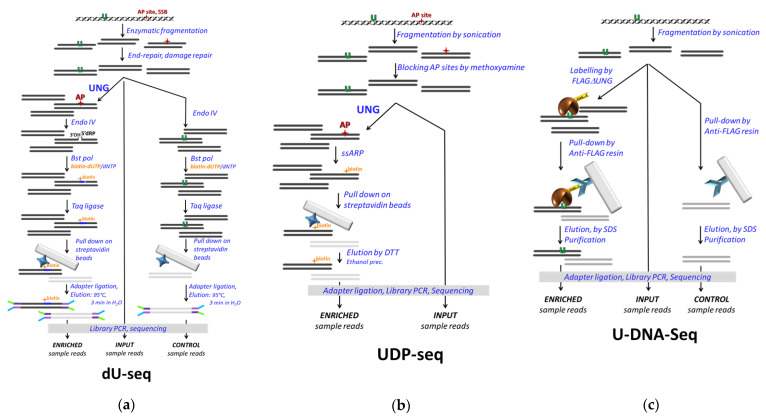
Pull-down-based methods for genome-wide mapping of U-DNA. (**a**) dU-seq. Pre-existing AP sites (red star) and single strand breaks (SSBs) require in vitro repair before the combined UNG/ENDO IV reaction on uracil (green U) containing genomic DNA fragments. Bst polymerase (Bst pol) is used to add nucleotides utilizing biotin-labelled dUTP to the free 3′-OH end and to remove the 5′-deoxyribosephosphate (5′dRP). The biotin labelled and re-ligated fragments are pulled down by streptavidin beads. Sequencing results are compared to the control pull-down without UNG treatment. (**b**) UDP-seq. Pre-existing AP sites (red star) are blocked before the UNG treatment. Uracil-related AP sites are chemically labelled by an aldehyde reactive probe (ssARP) conjugated to biotin via a disulfide bridge-containing linker allowing efficient elution by dithiothreitol (DTT) upon pull-down. Sequencing results of pull-down sample is compared to the genomic input. (**c**) U-DNA-Seq. Uracils in genomic DNA fragments are specifically labelled by the FLAG-tagged ΔUNG sensor (FLAGΔUNG) and pulled down using anti-FLAG agarose. Sequencing results of pull--down sample is compared to the genomic input, and non-specific binding is estimated by a control mock pull-down.

A novel genome-wide uracil detection method, dU-seq, was also combined with the pull-down technique [112] (Figure 5a). This method applies an enzymatic cascade including *E. coli* UDG to convert genomic uracils into AP sites that are cleaved by ENDO IV, and the gaps are resynthesized by Bst DNA polymerase in the presence of biotinylated nucleotide triphosphate. The biotinylated DNA fragments then pull down on streptavidin beads, where the Y adapter for sequencing is ligated before the elution is conducted for 3 min at 95 °C in distilled water. The eluted DNA fragments are amplified by PCR and sequenced by Illumina. Prior to the enzymatic treatments, repair of AP sites, ssDNA breaks, and ssDNA ends were performed. The input and the enriched samples were sequenced, and peak calling was performed by model-based analysis of ChIP-seq (MACS2) software. Only peaks uniquely present in the pull-down versus the control were considered in the consequent analysis. Uracil enrichment within the centromeres was reported, which was also confirmed to some extent by independent methods including pre-digestion Excision-seq, LC-MS/MS, and 3D-PCR.

The UDP-seq method, another DNA-IP-seq application, is quite similar to dU-seq except that the introduction of the biotin label to the uracil sites is performed in a non-enzymatic chemical reaction [86] (Figure 5b). The alkoxyamine moiety of the commercially available reagent EZ-Link Alkoxyamine-PEG4-SS-Biotin (ssARP) (Thermo Scientific) covalently labels the opened ring of the base-free deoxyribose at the AP site. The S–S bridge allows efficient elution by reducing agents such as dithiothreitol (DTT). Chemical blocking of pre-existing AP sites that otherwise could interfere with uracil detection is necessary. Application of UDP-seq in bacterial systems showed, on one hand, that upon dUTPase and UNG deficiency, the elevated uracil incorporation occurs mostly at the replication origin. On the other hand, the ectopically expressed APOBEC3A (A3A) catalyzed cytosine deamination patterns were addressed in *E. coli*, where both UDGs are mutated. The control pull-down without UNG treatment to check for non-specific binding was omitted (Figure 5b vs. Figure 5a), but control samples were introduced: active A3A-expressing cells were compared to either inactive A3A or empty plasmid-containing cells. In the data analysis, peak calling was performed with MACS, and a normalized differential coverage (NDC) for 100 bp moving window was calculated. A uracilation index (UI) was introduced to measure the frequency of TC to TT transitions specific for the A3A, and a preference of A3A activity was detected for the lagging strand during replication, as well as in short hairpin loops, tRNA and rRNA genes, and in the 5′ termini of some protein coding genes.

In both dU-seq and UDP-seq, numerous enzymatic/chemical steps are involved, resulting in a complex arrangement with multiple potential pitfalls. Additionally, abasic sites independent from uracils need to be carefully considered. Moreover, sticky DNA ends could influence the polymerase-based labelling approach in dU-seq. Accordingly, both dU-seq and UDP-seq are based on well-established experimental setups and take advantage of the highly efficient biotin–streptavidin pull-down system. Processing and the interpretation of the NGS data involve several critical issues that will be detailed in Section 3 and Section 4.

The most recent pull-down-based method, U-DNA-Seq, employs U-DNA-specific binding of the FLAG-tagged ΔUNG sensor (already described above) to pull down uracil-containing genomic DNA fragments. As such, it is a more direct method and involves less complex steps than dU-seq and UDP-seq (Figure 5c) [91]. It is independent from the efficiency of different enzymatic/chemical reactions used in dU-seq and UDP-seq and relies on the specificity and affinity of the interactions between ΔUNG and U-DNA, and the anti-FLAG antibody and the FLAG-tag, respectively. In the published paper, U-DNA-Seq was applied in the human cancer cell line HCT116, and its mismatch repair proficient version, where the UNG inhibitor UGI was stably expressed [91]. The effects of two thymidylate synthase inhibitory drugs, 5FdUR and RTX, on the genomic uracil content and its distribution were addressed. Using their own analysis pipeline, remarkably high reproducibility among replicates (cf. Supplementary Materials in [91]) was presented even when results were compared to relevant samples from the published dU-seq data remapped and re-analyzed by the same pipeline.

The experimental design, the applied biological models, and data analysis, as well as basic conclusions of the studies using the above described pull-down-based U-DNA mapping methods are summarized in Table 1.

It is interesting to consider if a similar pull-down coupled sequencing method could be developed based on UdgX. Sequencing over the crosslink might be challenging, although may not be impossible to solve. Considering that UdgX has strong preference towards uracils in ssDNA [85], and once it binds to it, other UDGs cannot initiate its conversion to AP sites anymore [92], such an approach could provide selective and safe enrichment of otherwise more vulnerable uracil-containing ssDNA fragments directly from cells. Moreover, high throughput analysis of the crosslinked peptide–DNA fragments by mass spectrometry could also provide single base resolution data wherever it is interesting.

All of the methods described above potentially capable or used for genome-wide mapping of uracil moieties have advantages and limitations. The Excision-seq (pre- and post-digested versions), dU-seq, snAP-seq, UDP-seq, Nick-seq and U-DNA-Seq were used for genome-wide studies at different levels of genomic complexity, and for different biological samples with markedly different level and origin of uracil bases. Thus, the evaluation of the sequencing results as well as drawing conclusions requires appropriate considerations.

## 3. Factors to Consider in Analysis of NGS Data to Maximize Relevant Information While Avoiding Over- or Misinterpretation

We have presented a wide overview of genomic uracil detection methods applied to provide insights into profoundly different biological problems. Appropriate data processing and analysis are essential to avoid over- or misinterpretation. To promote the research interest in this field, we hereby discuss some potential pitfalls of data analysis that can easily lead to overinterpretation of the results. Carefully designed and performed experiments with appropriate controls are as crucial as the appropriate choice of analysis pipeline, including double-checking all calculation steps because even the widely used software often suffers from hidden bugs (as it is reflected in the open issues on GitHub).

Analysis of NGS data that are designed for mapping a feature (i.e., uracil) to the genome relies on an initial alignment of the short sequencing reads to a chosen reference genome set. Importantly, none of the reference genomes are fully relevant for any particular biological sample due to the unique character of the individual genomes with multiple polymorphisms including copy number variations. The current human reference genome (Genome Reference Consortium Human Reference 38 (GRCh Build 38 or hg38 in GeneBank), https://www.ncbi.nlm.nih.gov/grc/human, accessed on 7 April 2021) actually exists in multiple and somewhat different sets. These include not only the main chromosomes, but different sets of hundreds to thousands extra scaffolds, such as unlocalized (UnChr…) or unplaced (Un…) genomic segments, established genomic alterations (ALT…), viral, and other decoy sequences. Moreover, critical segments might or might not be masked in these sets, where certain gaps also occur. Therefore, the choice of the reference genome set can influence the resulting read alignments. Alignment files usually cannot be shared in databases such as Gene Expression Omnibus (GEO); therefore, unequivocal definition of the used reference genome set is crucial in all publications to promote reproducibility.

The high frequency occurrence of repetitive regions in the human genome unfortunately interferes with mapping of many short sequence reads. Ambiguously, mapped reads are indicated with zero mapping quality value within the alignment files but are not automatically excluded from downstream analysis. There is always a trade-off between loss of information and allowing the possibility of incorrect alignment. As we have already demonstrated [91], applying a sample specific blacklist and filtering for ambiguously mapped reads might be a solution to avoid misinterpretation of the data. A blacklist appropriate for the given biological sample should be defined by low mappability and by ultra-high signal (UHS) regions in addition to the otherwise quite narrow universal blacklist recommended by the ENCODE [121].

Certainly, if the research question focuses on such critical low mappability regions, blacklisting the whole region of interest is not an option. In such cases, application of strict measures and/or alternative approaches is required. The human centromeres are among the best examples for such critical regions with megabase-size higher order repeats consisting of several short alpha-satellite sequences [122], for which model sequences have already been implemented in the GRCh38 reference genome [123]. Although these models allow alignments to the centromeres, it should be done with extra care. In the dU-seq method [112], although the whole experimental design reflects supreme care and accuracy, the details of bioinformatic analysis are under-documented and may suffer from some artefacts. Namely, ambiguously mapped reads were most probably not excluded from the downstream analysis that was completely based on the results of the peak calling. In a highly repetitive region with potential massive copy number variations, the read alignment necessarily suffers from high amount of ambiguously mapped reads and UHS segments. Under these circumstances, local increases and decreases in the read coverage are not reliable; hence, peak calling algorithms can certainly detect more false positive peaks. Therefore, peak calling is not an appropriate approach in this case. Instead, a chromosome-wise comparison of the normalized read counts (RPKM) within the centromeres versus the non-centromere regions would have been more reliable, which was performed in the evaluation of the complementary Excision-seq experiment, but not for the dU-seq data. Based on the distribution of detected peaks in dU-seq data, a high centromeric uracil enrichment was suggested (up to 20–25-fold in wt and *ung*^−/−^ HEK293T, reported in Figure 5c in [112]). This result was confirmed to some extent by LC-MS/MS quantification (2.2-, 5- and 7-fold centromeric uracil enrichment in wt HEK293T, K562 and WPMY-1 cells, Figure 3e in [112]), and also by Excision-seq (approximately 1.6-fold centromeric uracil enrichment in K562 and WPMY-1 cells, Supplementary Figure S7 in [112]). The much lower centromeric uracil enrichment detected by Excision-seq and LC-MS/MS quantification as compared to dU-seq was contributed to the limited sensitivity of the method and with lower efficiency of the centromeric DNA isolation protocol, respectively. The dU-seq data were reanalyzed and this issue was discussed in detail [91].

To judge the reliability of any analysis, the best measures are the correlations among the replicates for which different statistical tests are available. We suggest that figures in the main text should represent merged data from the replicates; however, the same analysis for the individual replicates and correlation measures should definitely be reported at least as supplementary materials. In case of U-DNA-Seq performed on HCT116 cells, such analysis showed that the called peaks are less reproducible and have lower description value for the genomic uracil patterns as compared to the proposed broad genomic regions derived from log2 enrichment tracks [91]. Individual replicates have an inherent variance due to natural alterations among biological samples and the limited depth of the sequencing as compared to the “depth” of the actual DNA sample. If genomic DNA is sequenced from a cell line, the starting material typically represents about 10 million diploid cells, while the average sequencing depth is only 6–7-fold in the case of typical 20 Gb, ~130 M raw reads, 150 PE Illumina sequencing. Depending also on the original genetic heterogeneity of the given cell line, such sampling in the sequencing itself leads to somewhat limited significance of the results. This can be controlled by the statistical analysis of the individual replicates.

## 4. Single Base Resolution—When Is It Truly Relevant?

The recent literature in the field reflects high demand on gaining base-resolution data on genomic distribution of certain DNA damage and base modifications [72,73]. Among potential uracil-DNA sequencing approaches, pre-digestion Excision-seq, snAP-seq and Nick-seq are potentially capable of identifying the exact position of individual uracils.

From these methods, only pre-digestion Excision-seq was used directly for mapping genomic uracil in *E.coli* and yeast models with UNG and dUTPase deficiency, where a high rate of thymine-replacing uracil incorporation is expected [110]. Although the capability for single-base detection of the pre-digestion Excision-seq was demonstrated, it was not really exploited in these systems because of the stochastic distribution of the incorporated uracils. Excision-seq was further used for 10 kb-size HIV genome where a thymine-replacing incorporation mechanism was also suggested, and the single base resolution was not relevant to describe the relatively homogeneous distribution of uracils [100]. The only case when Excision-seq was used in the context of complex vertebrate genomes was a validation experiment for the dU-seq data, where a modest (~1.6-fold) centromeric enrichment of reads was observed as compared to the genomic average [112]. The potential benefit of the single-base data was not addressed in this study, i.e., there was no attempt to extract consensus sequence motifs around these uracils; however, the conclusion assumes some regulatory mechanisms behind the proposed centromeric uracil enrichment [112].

In contrast, snAP-seq was used for the mapping of hmU sites with truly single base resolution in the *L. major* genome, where hmUs could be selectively converted to AP sites using SMUG1 uracil-DNA glycosylase [103]. A considerable number (3200) of sites were identified with high confidence within four replicates that were in good agreement with the previously measured extremely high frequency data (~100/10^6^ bases) in the 33 Mb genome and also with the previously reported low resolution genome-wide data [124]. It was confirmed that these sites corresponded to hmU and not U or other U derivatives by using a negative control experiment using UNG (not capable of excising hmU). In *L. major*, hmU was shown to serve as a precursor of epigenetic mark base J involved in transcription termination; hence, it is supposed to be introduced to the genome most probably by dedicated enzymes [117,118,119]. This amazing performance was further rationalized by the analysis of the sequence context that revealed some preferences indicative for an enzymatic reaction by which hmU is introduced to the *L. major* genome. In contrast to this representative single-base resolution detection in *L. major*, the same snAP-seq method failed to detect high confidence AP sites in HeLA cells, even upon silencing APE1 [103]. Based on this experience, it was concluded that there are no well-defined APE1 substrate AP site positions within the genome, but wider stretches were found where the probability of AP site occurrence was higher. The genome-wide co-localization analysis of these regions with known genomic features revealed new information about typical genomic location where APE1 is active (promoter, UTR, and exon segments); however, a lower resolution method would have also been appropriate to provide the same information.

This study excellently demonstrated that the single-base resolution detection has relevance only in those cases where uracils are introduced by targeted enzyme reactions to preferred positions that should then also imply some regulatory role. Hence, application of such techniques can be extremely beneficial to explore new biological contexts, where uracil might serve as an epigenetic signal. If such properties are assumed, it is an obvious opportunity to check the immediate sequence context of the mapped sites searching for conserved recognition motifs. Detection of such motifs on the one hand strengthens the reliability of the base-resolution distribution data, while on the other hand it might provide a strategy to identify new enzymes responsible to introduce uracil into the specific genomic loci.

In the case of genomic uracil, intriguing questions arise on the widely studied field of cytidine deaminases; AID/APOBECs involved in basic processes of acquired immunity and in antiviral responses. The sequence preferences and other regulatory factors in the targeting of AID/APOBECs were extensively addressed by reporter systems involving mutational analysis [125] and even a base-resolution U-DNA sequencing technique (LM-PCR [98]). However, genome-wide studies were performed only with indirect mutational analysis [25], or with the lower resolution UDP-seq within the context of a limited-size bacterial genome [86]. To the best of our knowledge, no genome-wide and single-base resolution study has yet been published in this field.

Notably, even with a lower resolution genome-wide method, it is possible to gain relevant new information if uracils are introduced in a more targeted way to the genome. The UDP-seq method was used to analyze genomic DNA from APOBEC3A expressing *ung*^−^/*mug*^−^ *E. coli* (representing a veritable lack of UDG function). Results showed somewhat different patterns for the wt APOBEC3A as compared to the controls (inactive APOBEC3A or empty vector). Focusing on the known minimal target motif of APOBEC3A (TC) and calculating a uracilation index (UI) for each TC in the genome revealed that APOBEC3A acts preferentially on the lagging strand [86].

It is important to consider that both spontaneous cytosine deamination and uracil incorporation through dUTP pool increase are basically stochastic processes. In these cases, single base resolution may have true impact only in single-cell sequencing, because the actual positions of uracils are expected to be variable in every single cell. However, it is well demonstrated that even in these cases, the genomic uracil distribution is not fully random, but is influenced by several circumstances. In the case of drug-induced uracil incorporation, the main determinant might be the local rate of ongoing DNA synthesis, either replicative or repair-related [91]. Genomic patterns of uracil enrichment upon the effect of two TS inhibitory drugs indicated a strong correlation with replication timing and, to a lesser extent, with the more actively transcribed euchromatin. Interestingly, clear differences were shown between the uracil enrichment patterns induced by the two drugs. In contrast in the non-treated cells, the low amount of uracils showed primarily heterochromatic enrichment [91]. In the case of spontaneous cytosine deamination, altered accessibility of DNA within differently packed chromatin structures (from heterochromatin to the actively transcribing genes), or in double-stranded/single-stranded segments [126], and the diverse protein bound states (nucleosomes, transcription factors, DNA manipulating enzymes, etc.) might also influence the local frequency of deamination reactions. Similarly, the genome-wide distribution of distinct UDGs and other repair enzymes including polymerases must have a strong impact on the actual genomic uracil patterns. Therefore, a statistical approach might provide higher descriptive value while avoiding misinterpretation of the data. Identifying genomic regions (or peaks) where uracil can occur with higher probability, and comparison of these segments to other known genomic features might shed light on the underlying cellular processes.

## Figures and Tables

**Table 1:** Summary of studies based on dU-seq, UDP-seq and U-DNA-Seq. Abbreviations are as follows: *ung*, *dut*, *mug*, and *hmlh1* are genes encoding UNG, dUTPase, double stranded U-DNA specific UDG of *E. coli*, and human MLH1, respectively. MMR: mismatch repair, A3A and A3A*: APOBEC3A and its inactive mutant, EV: empty vector, NDC: normalized differential coverage, UI: uracilation index.

	dU-Seq	UDP-Seq	U-DNA-Seq
Genome	human	*E. coli*	human
Gene deficiency	*ung*^−/−^ wt	*ung*^−^/*dut*^−^ vs. wt, *ung*^−^/*mug*^−^	*hmlh1*^−/−^ Restored MMR
Transgene	- *ung* UDGs	- A3A, A3A* EV	*ugi* -
Treatment	- 5FdUR	- -	5FdUR, RTX -
Data pre-processing	Trim, Align	Trim, Align, Filter	Trim, Align, Blacklist, Filter
Enrichment analysis	Peak calling	Peak calling, NDC, UI	Coverage, log2 ratio, broad regions
Conclusion	Centromeric enrichment	*ung*^−^/*dut*^−^: replication origin A3A in *ung*^−^/*mug*^−^: lagging strand, hairpin loops, tRNA genes	Heterochromatin, upon treatments: shifted towards early replicating and active euchromatin
